# The voices of family caregivers of seniors with chronic conditions: a window into their experience using a qualitative design

**DOI:** 10.1186/s40064-016-2244-z

**Published:** 2016-05-14

**Authors:** Suzette Brémault-Phillips, Jasneet Parmar, Melissa Johnson, Arlene Huhn, Anna Mann, Victoria Tian, Lori-Ann R. Sacrey

**Affiliations:** Department of Occupational Therapy, Faculty of Rehabilitation Medicine, University of Alberta, 2-64 Corbett Hall, Edmonton, AB T6G 2G4 Canada; Department of Family Medicine, University of Alberta, Suite 205 College Plaza 8215 - 112 St., Edmonton, AB T6G 2C8 Canada; Specialized Geriatrics Program, University of Alberta, Suite 205 College Plaza 8215 - 112 St., Edmonton, AB T6G 2C8 Canada; Alberta Health Services, Home Living, Edmonton Zone Continuing Care, 10216-124 Street, Edmonton, AB T5N 4A3 Canada; Network of Excellence in Seniors Health and Wellness, Covenant Health, c/o Grey Nuns Community Hospital, 416 St. Marguerite Health Services Centre, 1090 Youville Drive West, Edmonton, AB T6L 0A3 Canada; Network of Excellence in Seniors Health and Wellness, Covenant Health, c/o Grey Nuns Community Hospital, 416 St. Marguerite Health Services Centre, 1090 Youville Drive West, Edmonton, AB T6L 0A3 Canada; Client Services and Programs, Alzheimer Society of Alberta and Northwest Territories, 10531 – Kingsway Avenue, Edmonton, AB T5H 4K1 Canada; Alberta Caregivers Association, 10310 - 56 St NW, Edmonton, AB T6A 2J2 Canada; Faculty of Medicine and Dentistry, University of Alberta, 2J2.00 WC Mackenzie Health Sciences Centre, 8440 112 St. NW, Edmonton, AB T6G 2R7 Canada; Department of Pediatrics, Faculty of Medicine and Dentistry, Autism Research Centre (E209), 10230 - 111 Avenue Glenrose Rehabilitation Hospital, Edmonton, AB T5G 0B7 Canada

## Abstract

**Background:**

Family caregivers are the backbone of the healthcare system. Over time, caregiving takes a tremendous toll on the caregiver. This is particularly true for caregivers who (1) provide >21 h of care/week, and/or (2) support those experiencing depression, cognitive decline, aggressive behaviours, and life-limiting conditions requiring complex care. Many caregivers face deteriorating physical and mental health, social isolation, family conflict, and job loss. Caregivers often have little energy or time to access resources and their experiences with the healthcare system, healthcare professionals and service agencies can either buoy them through challenging times, or contribute further to their distress.

**Objective:**

This project aimed to hear the voices of family caregivers; their challenges, struggles, joys, and motivation for persevering through hardship, as well as their recommendations regarding education, resources, and supports that might enhance their resilience.

**Methods:**

This community engagement research project utilized an ethnographic, qualitative approach involving three, 2-h focus groups that were analyzed using thematic analysis.

**Findings:**

Caregivers identified barriers to resilience, including demands on their time, changing roles and responsibilities, challenges of learning about medical conditions, their own emotional responses, financial strains, changing family dynamics, and personal health. Caregivers also identified several facilitators to resilience, including motivations for caregiving, sense of purpose and validation, spirituality, emotional experiences, and coping strategies.

**Conclusion:**

Caregivers recommended that educational opportunities, including increasing health care providers education concerning dementias, increased access to resources, system navigators, financial supports, political advocacy, and a more responsive caregiver centered system would support family caregiving.

## Background

Canada’s population is aging and the role of family caregivers is becoming increasingly important in providing necessary support. It is anticipated that the proportion of individuals aged 65 and over will increase from 15 % up to 28 % by 2063 and those 80 and over will grow from 1.4 million up to 4.9 million by 2044, when they will account for 10 % of the population (Bohnert et al. 2015). More Canadians are also living with chronic diseases, with 33 % of those aged 80 years and older having four or more chronic conditions, including dementia (Butler-Jones 2010). Individuals living with dementia in 2011 accounted for over 700,000 Canadians aged 65 years and older and may increase to 1.4 million by 2013 (Alzheimer Society of Canada 2012). Family and friends are often relied upon to help these individuals continue to be active and remain connected to their communities (Eales et al. 2015).

Family caregivers, recognized as the backbone of the health care system (Butler-Jones 2010; Kitts 2012; Sinha 2012), provide informal unpaid care (World Health Organization 2012). In Canada, the costs of unpaid caregiving have recently been estimated at $25 billion (Hollander et al. 2009).  A 2012 survey (Sinha 2012) found that, of the 8.1 million Canadian carers (28 % of population), 44 % were between the ages of 45 and 64 years, 10 % provided more than 30 h of support a week, 60 % continued to work while providing care, and 25 % were simultaneously caregiving and child rearing. Furthermore, approximately 50 % of family caregivers cared for seniors with health conditions (Turner and Findlay 2012) and close to half a million supported a person with dementia (often a parent or in law) (Eales et al. 2015). The overwhelming majority of caregivers (89 %) offered support for a minimum of one year, with 50 % doing so for at least four years (Sinha 2012). The care provided by family members includes a variety of activities. Most frequently, they provided transportation (73 %; e.g. running errands, shopping, attending medical appointments, participating in social events), performed tasks in the care partner’s home (52 %; e.g., preparing meals, cleaning, laundry), and assisted with house maintenance or outdoor work (45 %). Support for personal care and medical treatments varied, with caregivers offering weekly (66 and 63 %, respectively) or daily (both 34 %) support. Additionally, over 88 % of caregivers provided emotional support, including spending time and talking with the care partner, and 96 % visited or called to ensure that the care partner was okay (Sinha 2012).

Supporting family caregivers has become a national public health priority given their essential role in the healthcare system (Hollander et al. 2009). The Health Council of Canada’s (Kitts 2012) “seniors in need, caregivers in distress” report identified the need to support caregivers and recognize their indispensable contribution in the sustainability of the health care system. Caregivers require support to foster resilience (adapt well in the face of adversity) and ensure that they can continue to provide care while maintaining their own wellbeing. While the majority of caregivers report being in good, very good, or excellent physical and mental health, caring can take a toll on caregivers and leave them increasingly overburdened (Butler-Jones 2010; Eales et al. 2015; Kitts 2012; Sinha 2012). Caregivers are at an increased risk of significant physical, emotional, and financial burden if: (1) they provide more than 21 h per week of care; (2) care for persons with depression, cognitive decline, behavioural change; or (3) care for persons with terminal conditions (Butler-Jones 2010; Kitts 2012; Sinha 2012; Hollander et al. 2009; Canadian Institutes of Health 2009). The resultant stress can lead to deterioration of their health, social isolation, loss of income, and family conflict (Kitts 2012; Stajduhar et al. 2010; Dumont et al. 2009). The strain on family caregivers is anticipated to intensify as a result of the aging population (Eales et al. 2015; Smetanin et al. 2010; Dudgeon 2010).

Inclusion of evidence-informed supports for family caregivers should be an important part of any regional, provincial, national, or international strategy (Parmar et al. 2014). There is, however, a gap between what is known of the caregiver experience and what is most likely to offer support. A better understanding of the experience of caregivers and ways to foster their resilience is needed. This project aimed to hear the voices of family caregivers—their challenges, struggles, joys, and motivation for persevering through hardship, as well as their recommendations regarding education, resources, and supports that might enhance their resilience. To that end, and as part of a pre-conference activity to a CIHR planning conference “Supporting Family Caregivers of Seniors” held in Edmonton in April 2014, three 2-h focus groups were held with family caregivers residing in the Edmonton area. The anticipated outcomes of the focus groups included acquiring a greater understanding of the caregiver experience, identifying what is needed to improve service provision and caregiver support, and research priorities. The outcomes of the focus group discussions were largely centred on the experience of caregivers of seniors with complex needs. The purpose of this paper is to: (1) describe the experiences, challenges, facilitators, and rewards identified by family caregivers of persons with complex needs, and (2) outline recommendations regarding education, resources, and support to increase caregiver resilience that could inform research priorities.

## Methods

This community engagement research project utilized an ethnographic, qualitative approach involving focus groups and thematic analysis.

### Participants

Participants were invited to attend one of three focus groups at the invitation of the Alzheimer Society—Alberta and Northwest Territories and the Alberta Caregivers Association. Flyer/poster solicitation and invitations were utilized to recruit participants who were known to the organizations. Overall, 23 family caregivers of persons with complex needs residing in the Edmonton area participated in one of three focus groups each comprised of 7–8 caregivers.

The family caregivers indicated that they supported between 1 and 4 individuals with complex needs (hereafter, ‘care partners’): 1 care partner (n = 16), 2 care partners (n = 5), and 4 care partners (n = 2). Years of caregiving ranged from 1 to 12 years, with an average of 5.2 years per care partner. Female focus group participants (n = 15) provided a total of 138.5 years of caregiving to husbands (n = 7), fathers (n = 4), mothers (n = 8), fathers-in-law (n = 3), and mothers-in-law (n = 3)—an average of 5.54 years per care partner. Male participants (n = 8) provided a total of 38.5 years of caregiving to wives (n = 4), mothers (n = 3), and fathers (n = 2)—an average of 4.28 years per care partner. The majority of focus group participants provided support to individuals over age 65, with primary diagnoses of dementia (n = 25), cancer (n = 3), Parkinson’s disease (n = 2), unspecified (n = 2), stroke (n = 1), and brain injury (n = 1).

### Focus groups

Three 2-h focus groups were held with family caregivers on April 2nd and 3rd, 2014 at the Alzheimer Society and on April 4th, 2014 at the Alberta Caregivers Association in Edmonton. The focus groups were co-facilitated by 2 or 3 research team members with a minimum of 7 years experience working with seniors with complex needs and their caregivers. Focus group questions (see Table 1) captured various aspects of their experience, supports, and recommendations.

**Table 1:** Focus group questions for family caregivers

Focus group questions	
(1) What current resources do you rely on to help support you in your role as a family caregiver?	
(2) What resources are currently not available to you that you think would really help you in your role as a family caregiver?	
(3) Do you feel you have all the necessary knowledge and skills required to provide care to your family member? If not, what are the areas where you would like more knowledge and skills?	
(4) Where do you go to or would you go to get more knowledge, skills and/or resources? (e.g. Internet? Printed materials? Telephone help lines? Group meetings? One-on-one in-person support?)	
(5) What is most challenging to you in your role as a family caregiver?	
(6) What is most rewarding to you in your role as a family caregiver?	

Focus group discussions were audio-recorded, professionally transcribed, and entered into NVivo 9 for coding and analysis by members of the research team (SBP, MJ, VT, JP). Standardized coding techniques, using an adapted version of Roper and Shapira’s (Roper and Shapira 2000) approach, were utilized to identify patterns and emergent themes (Roper and Shapira 2000). Notes collected during the focus groups tracked themes as they emerged throughout the discussion. Notes from the second day of focus groups did not yield new themes, thus resulting in data saturation. To ensure the integrity of the research process, four aspects of trustworthiness, detailed in Lincoln and Guba’s model (Lincoln and Guba 1985) were addressed throughout the analysis processes: (1) promoting *credibility*, (2) promoting *transferability*, (3) ensuring *dependability*, and (4) *confirmability* (through triangulation with stakeholders). Authors met to discuss, validate, and come to consensus around themes and five focus group participants validated the findings. The study received ethics approval from the University of Alberta Health Research Ethics Board-Health Panel.

## Findings

Themes that emerged from the data analysis included barriers, facilitators, and recommendations in support of caregivers. These findings are presented below.

### Caregiver experience: barriers to resilience

Many caregivers experience significant stress due to shifting roles and responsibilities, demands on their time and resources, financial instability, changing family and social dynamics, deterioration of health, emotional strain, and challenges working with the healthcare system. An elaboration of these themes with supporting quotes can be found in Table 2.

**Table 2:** Caregiver experience: barriers compromising resilience

Themes	Findings and supporting quotes
1. Caregiver	Caregivers reported barriers to resilience associated with demands on their time, changing roles and responsibilities, challenges of learning about medical conditions, their own emotional responses, financial strains, changing family dynamics, and challenges with their personal health
1.1 High demands on time and energy	Caregiving becomes increasingly demanding as the care partner becomes more mentally and physically compromised. Provision of occasional support (e.g., doctor appointments) progresses to support for everyday activities (e.g., dressing, bathing, medication administration). *“Caregiving is a 24/7 job”* Caregivers sacrifice time and energy to respond to the care partner’s needs, leaving little to no time to tend to their own needs, or those of other friends and family. Their own health can easily be compromised. *“It’s just simply a matter of time and energy”*
1.2 Shifting roles and responsibilities	Caregivers often assume a more “*parental*” role, as they help with daily activities, such as dressing, feeding, and maintaining the person’s hygiene. *“I do absolutely everything at home, I mean you name it I do it”* Some care partners lack insight into their limitations (e.g. managing finances), adding a tension-filled burden. As caregivers provide support, including responsibility for *managing medical concerns* of care partner, the caregiver’s familiarity makes them a rich resource for monitoring treatment and identifying areas of concern. *“I have to go in with him now, if he has a doctor’s appointment or dentist appointment I have to go in with him to make sure the communication is there and he doesn’t get confused and things naturally get accomplished”*
1.3 A new world - understanding medical infor-mation/jargon	Caregivers often feel overwhelmed by their care partner’s diagnosis, do not understand the diagnosis, and may not have the ability to search for answers. Without the necessary information, they cannot be effective supports and advocates. *“I need knowledge about what they’re doing for him and to him so I can be with him and know something about why he’s doing what he’s doing”*
1.4 Assumption of decision-making responsibilities	Decline in the care partner’s cognitive capacity can result in caregivers taking on responsibility for financial and legal decision-making. Caregivers who are not legal guardians may be unable to effectively advocate for the person. *“I got a call from central booking; they won’t talk to me because I do not have power of attorney. I find that a big deal because I can’t do anything, I am stuck, I am so stuck”* and *“I’m just really frustrated and I find* – *I’m trying to think of the word, like I feel so stuck because I know there is stuff out there that I should be able to access, but because the way he is, I can’t do it, I don’t have the power of attorney”* Caregivers, even if they are legal guardians, noted that they are not always updated regarding the care partner’s care. *“Even though you’ve signed that paper, it doesn’t necessarily mean that… you’re told enough”*
1.5 Changing family dynamics	Changing relational dynamics occur throughout the caregiving process - arguments, social isolation, heightened feelings of resentment and anger may result. *“My daughter hasn’t come around since before Christmas, and she only lives 5* *min away. She hasn’t even called; she says, ‘mom I don’t know why you do this, put him in a home’”* Differences of opinion arise in families regarding caregiving decisions. *“I was accused by family members that I was depriving her of her religious beliefs”* and *“I think what we all are here is decision makers and we have to make those decisions and in some cases the decisions we make sometimes conflict with family”* Caregivers contend with competing priorities and lost opportunities, particularly those with young families. Caregivers try to balance caregiving with childcare and may feel they are “*being pulled in different directions.*” *“I had two little kids of my own to chase as well. So you really feel like you sacrificed and it starts* – *sorry, starts out as a good deed and it turns into a really big burden”* and *“So I’m constantly seeking ways to make up for missed moments”*
1.6 Emotional experience	Caregivers can experience a range of negative feelings that can be barriers to resilience: Caregivers often feel that they are not in control and are always behind. *“It’s just like it’s two steps forward and one back all the time. I’ve never been ahead of the darn ball; I’m always catching up. I’m never feeling like oh great I’ve got this under control, I never do”* Caregiving was described as a continuous grieving process. This is especially true for caregivers of spouses, as the decline can signal loss of their closest companion. *“Loss and grief. My husband and my relationship is not the same as he becomes sicker, it’s a caregiver/recipient relationship and so you lose companionship and friendship”* Caregivers may also mourn the loss of their caregiving role at the end of the journey. *“It becomes a role, … purpose in life, and even though it was a burden, when you remove that role or purpose, they feel a loss”* Uneven sharing of caregiving responsibility can lead to resentment and anger between caregivers. *“My brothers both have families and they basically both cut lines in the sand that you know like “we can only do so much and our primary responsibility is to our own families.” …They’re not quite doing as much as they could or should and I have some resentment”* and *“I just feel like I am carrying this burden and I’m the only child here out of 5.* *And I feel like I’ve been given a life sentence”* Caregivers expressed feeling guilty and selfish for wanting a break or asking for help. *“I feel like a lot of guilt because I feel like I should be doing more”* Guilt also becomes salient when caregivers place care partners into a care facility. *“I had to place him. So it was a tough year. And I had so much guilt. I just … it was … I just felt like it was all my fault”* Caregivers report feeling alone in their struggle. *“What I find is going through this journey, people start staying away because this is what you live especially when you’re living it twenty*-*four/seven”* and *“Quite often you’re alone. There are a lot of family dynamics. Friends back away”* Witnessing the care partner’s decline forces caregivers to confront their own mortality, and question who will look after them as they decline. *“I look at myself, I’m starting to think what’s going to happen to me when I get older? I don’t have kids. I don’t have, I guess, a support group there. So I’m kind of in fear if this is going to happen to me”*
1.7 Mental and physical exhaustion	Caregiving can seem endless, take an immense amount of time and energy, and pose a seemingly insurmountable challenge. Caregivers can find themselves feeling “*burnt out*”, “*bone weary tired*” as if “*they have not slept for ages,*” and “*at the end of their rope.*” *“I said desperation, exhaustion total mental, physical, everything, like I just can’t do this anymore, but I have to - there’s nobody else”* and *“I realized I hadn’t slept for 5* *years because you’re always listening and waiting; you’re always just on the edge of sleep, you never actually sleep”*
1.8 Financial realities	The financial burden of caregiving can be quite significant and laden with unintended consequences on the caregiver’s own financial security. Some caregivers at the prime of their careers have “*had to give u*p” their job, and those who have lost their source of income had “*no emotional strength to job hunt.”* *“And so, you know, I also, you know, quit my job. You just get tired”* Many caregivers, already responsible for mortgage payments and the cost of childcare, experience further financial stress due to the burden of long term medical costs, facility fees, and private care expenses. *“Financial, I mean I was supporting 8* *years you know on my income, it was very difficult. Still had a mortgage, still had 3 children living at home, so there wasn’t anything there for me”* Some caregivers are forced to sell their homes, use their savings, or claim bankruptcy. *“So you’re left with this bill here and it’s sort of like, well you know it’s time for you to start using your RRSPs and paying for this. So now, you know, we use everything to pay for this”* and *“I’m on social assistance, I have no budget whatsoever to do anything sociable”* While financial assistance may be available, eligibility for funding depends on age and employment status. Younger caregivers may find themselves ineligible for financial compensation. *“I actually fall into the gap of not being old enough and still working and it’s not being retired, so I have to pay the $2,000.00 a month. So it’s quite a burden. On top of that, you know, we had to move at the wrong time when the financial downturn was on so, you know, we lost a lot of money moving to try and find the right healthcare”*
2 The System	Caregivers depend on healthcare professionals (HCPs) and the healthcare system to provide information regarding diagnoses, practical solutions regarding the patient’s medical concerns, and caregiver relief. While HCPs and the system offer caregivers guidance and resources, a variety of barriers were identified
2.1 Engagement with the healthcare system	Caregivers describe the system as harsh, rigid, policy-driven and unresponsive to each family’s unique set of challenges and needs. *“It sometimes feels the system is all about the system and it’s not about the people”* and *“It was all policy*-*driven. There was no compassion, no integrative support considering my dad’s condition”* Management pathways seem guided by standardized assessments and institutional mandates. Processes related to hospital discharge and placement assessments can be frustrating when caregivers perceive that test scores, discharge plans, and/or placements are not representative of the person’s capacity. *“They kept administering the MMSE test and the MoCA test to my dad. My father has a PhD as well and he kept scoring really high even though there were these challenges”* Navigating the system was reportedly akin to traveling through “a maze,” with information usually given in piecemeal format detached from the caregiver’s context. *“You find out this little bit of information and you have to take that and … go to somewhere else to find out the next part that you need and it was just an ongoing circle”* Caregivers reported having difficulty accessing resources, often finding valuable resources through internet searches or word of mouth. *“I learnt from a friend about the Alzheimer Society and when I sort of look back at dealing with the doctors, the professionals, it didn’t really come up”* Caregivers reported finding they are “*fighting and begging for help*.” Some caregivers prefer suffering in silence to enduring the frustration of asking for help, or being seen as the “enemy” as they advocate for better services and care. *“They make you go through so many hoops”* and *“I have to steel myself for a battle just to ask for anything”* People who suffer an acute onset, rapid decline or delirium may find themselves losing resources that they had worked hard to arrange (e.g. long-term placement), or unable to access much needed resources that may aid their recovery (e.g. therapy, programs). *“And every time she shows she’s either really, really down or really, really manically high…as she’s coming down you think ‘oh we can place her’ and then just before they place her, she drops down into the low”*
2.2 Engagement with healthcare professionals (HCPs)	Caregivers noted that inexperienced HCPs have inadvertently compromised patient care. *“Most people in the … healthcare system, you can say that they have dementia, they haven’t got a clue what it is. They think they do but they actually don’t”* Poor recognition of dementia symptoms can delay or prevent patients from accessing appropriate resources, timely and safe care, or placement opportunities. *“My mother had been seeing her GP. She’d been verbalizing her memory issues, her concern, at which he just said it is her getting old. He never gave her any medication. He didn’t test her. He didn’t send her to a facility”* A poor understanding of the dementia can also lead to unrealistic expectations of a client’s abilities (e.g. to live independently) and withholding of care. *“She’s dirty. She has outbursts. And they’re treating her like she is her own agent, that she is cognitively well and yet this is a dementia facility”* Without timely discussions of disease implications, caregivers feel left out, at a loss, and burdened by the weight of the diagnosis – unable to plan for the future and uncertain of how or from where to seek help. *“I do appreciate that they’re busy, you know, they’ve spent whatever, 10, 12* *years at university, but…”* When caregivers try to contribute to decisions, they at times feel dismissed and powerless despite the burden of responsibility they bear (including potential legal responsibility). *“Please talk to me at my level. Show me that respect”* and *“The system seems to be dismissive of caregiver input, the system is unresponsive, these two different worlds”*

*HCPs* health care professionals

### Caregiver experience: strategies and resources that facilitate resilience

Study participants identified many factors that support them as they provide care to their care partners. These included: personal attributes (motivations for caregiving, sense of purpose and validation, spirituality, emotional experiences, and coping strategies), relationship with the care partner, relational supports, and system supports. An elaboration of these themes with supporting quotes can be found in Table 3.

**Table 3:** Caregivers experiences: strategies and resources that facilitate resilience

Theme	Findings
1. Personal attributes	Caregivers identified personal attributes, including motivations for caregiving, sense of purpose and validation, spirituality, emotional experiences, and coping strategies that fostered resilience. *“I wasn’t sure either what I needed, you know, at the beginning of the process because this isn’t just about caregiving and my parents, it’s also about me and my personal journey”*
1.1 Personal motivations for caregiving	Family members may provide support for a variety of reasons, with several motivators being intrinsic factors, reciprocity, commitment, and behaviour modeling Caregivers noted being motivated to provide support out of love. *“I think I sort of learned a different dimension of love. I don’t know quite how to say that, but sometimes the greatest joy in my life is seeing my mom”* Caregivers of parents are strongly motivated to repay their parent’s love and dedication. Many spoke of a sense of duty, role of protector, or chance to care for their parents as their parents did for them in the past. *“My parents were awesome, they’ve been very, very supportive… were always there for me so it’s just natural that I would absolutely reciprocate, absolutely”* Spouses provide care out of an enduring commitment to their partner. *“I know my husband would go to the ends of the earth if it was me …”* and *“I just think this is my wife. I couldn’t abandon her”* Some caregivers noted the opportunity to model behaviours of compassion and commitment to their children, perhaps to encourage them to take care for them when they are older. *“I’m hoping that you know someone is going to be compassionate towards me, there’s a little bit of that, but it’s something they need to learn, so learning compassion”* Others hope that the experience is “*a time of growth*” for their children. *“It was a time of growth for my children, a terrible journey, but a real growth”*
1.2 The strength of the human-spirit	Caregivers spoke of the inner strength they draw on while providing support. *“Just knowing that I have found the inner strength to dig deep and find that compassion and whatever it takes to look after my parents…You know you’re doing the best for them and by them”* In times of struggle and loneliness, caregivers noted finding comfort in their spirituality. *“Internal resource…they’re my spirituality. They remind me that there’s a bright side of the deterioration and the dying and the grieving”* Some found strength in that which gives them meaning, purpose, or connection, or draw strength from prayer, their faith and relationship with God *“I do say prayers a couple of times a week. That got to be a habit that I was asking for help a couple of times a week for a long time”* For caregivers whose relationship with care partners are complicated by past abuse or estrangement, spirituality enabled them to forgive and heal. *“Part of my journey has been dealing with my parents who have been abusive* - *to now be advocating for them. I have been blessed that I have a sister who is a counsellor; I’m part of a pastoral team and it’s been very healing”*
1.3 Coping strategies	Caregivers employ a variety of coping strategies to regain control of what they can in their lives and accept that which they can’t (e.g. the care partner’s gradual deterioration). Such strategies help caregivers gain confidence and devise effective strategies to manage stress. Managing high expectations of themselves helps to decrease guilt and self-criticism. The recognition of their own limits leads to better self care, preventing caregiver burnout Self-acceptance: “*I’m doing the best I can and you know to me that helped get me through a lot because I knew I was doing the best I could and who can ask for more really”* Attitude of gratitude: *“I started to say thank you instead of help me and that has made a huge, tremendous difference to me”* Maintaining a sense of humour, attributing problematic behaviour to the illness, and focusing on the joys of caregiving helped them be resilient
1.4 Emotional experience	Caregivers can experience a range of positive emotions that can facilitate resilience. Caregivers described feeling strengthened when their care partner and others were appreciative of them, showed love, reassured them and recognized their efforts and commitment: Appreciated: *“My husband was always saying how much he loved me and how much he appreciated what I did and “without you I don’t know where I would be” and those kinds of things on a very regular basis. I felt very, very appreciated I really did”* Loved: *“Love, I mean in all kinds of ways and any sources including staff* - *they hug her and she smiles. Smiles are big and a generosity of spirit”* and *“When I’ve said, “I love you,” I have heard him mumble, “I love you too”* Reassured: “*I just feel good inside when I see that my dad is happy and well taken care of”* and *“I see a lot of kindness, I’ve seen a lot of good people… going through the system you know I’ve seen people maybe not skilled enough to do the job but kind enough to give a kiss and hug and I think that’s the one thing, I definitely …believe in people because I see what they do”*
2. Relationship with the care partner	Simple, yet profound, experiences with the care partner, gestures or words of gratitude, or a glimpse of the care partner’s momentary return to their former self, are enough to make the realities of caregiving worthwhile. *“But my reward is the times that I go there and I walk in the door, I see him walking down the hall and I’ll go up to him and I’ll just call him and I say “How are you today?” and he’ll turn around to me and look at me like I know who you are and he comes over and gives me a hug and a kiss. That does it for me”* As caregiving becomes routine, some find themselves enjoying the change in relationship with the care partner. The additional time spent deepens bonds, allowing caregivers to better know their partner, particularly when smiles were offered, laughter ensued, and memories were created. *“If I can get her to smile, my day is made”* and *“It’s just getting closer to my mom than I have been more so lately than the past 20, 30* *years”* and *“I’m getting to know my mom better than I have in that respect and, you know, I think it’ll just be quality time with her now”*
3. Relational supports: Family and friends	Friends and family are important sources of practical and emotional support. Family members alleviate burden by taking on responsibilities of caregiving (managing finances, housework tasks and transportation) for the benefit of the care partner and primary caregiver. *“My immediate family’s my biggest support. And they don’t get it like I do but they get me and they’re my normal, you know. If they can talk to me just about the good things, then it gives me the strength to carry on with all of this”* Spending time with friends and family can provide a sense of relief and normalcy. They offer companionship and support. Engaging with them in conversations and activities unrelated to caregiving provides an escape from caregiving responsibilities. “*They remind me that there’s bright outside of the deterioration and the dying and the grieving. You know, I’ve lost my mom. In fact, she’s not the mom she was. So they’re … they remind me of the way our lives used to be before all this happened”*
4. System supports/resources	Caregivers need to take time for themselves and be alleviated from some of the responsibilities
4.1 Respite care, day program or long-term facilities	Available respite services offer much needed help and are invaluable in providing momentary relief but caregivers are still responsible for the patients for the remainder of the day. *“I have lots of help and everybody thinks I have a piece of cake, but my shift runs from three in the afternoon till eight in the morning and on Saturdays from 11:30 to eight the next morning. So even though you get all the support, it’s still not a piece of cake”* The role of a caregiver continues when the care partner is placed into a facility. Caregivers regularly visit to provide companionship, bringing home cooked meals, bathing the person, or helping with exercise. In some cases, staff shortages and HCP stigmatism of certain diseases may lead to caregivers performing the roles of facility staff for the safety and benefit of the person. *“He’s now in the long term facility, but I still go and sort out … see what he needs and do a little bit of stuff for him because there are things that he needs that is not being addressed”*
4.2 Healthcare and community-based programs	Healthcare and community-based programs offer resources regarding education, yet system resources tend to focus on medical management of illnesses, and community resources on provision of supplementary services. The libraries and seminars organized by the health care system and community services disseminate valuable, user friendly information on the illness, its managements and coping strategies help patients and caregivers gain a better awareness and acceptance of the symptoms. *“It was nice to hear some of the language of caregiving and what it means to people and how important it is to look after yourself and keep yourself on track”* The sessions can offer caregivers with a safe space to express fears, loneliness, and frustrations. The ability to finally verbalize the emotional, physical and cognitive toll of caregiving can release tension and facilitate the healing process. *“The support groups have been the biggest help because you don’t feel alone and you feel like people actually care”*
4.3 Medical supports	Medical supports are irreplaceable regarding concerns from the person in care or caregiver. For many, a visit to a physician often marks the beginning of the caregiving journey. Physicians and allied healthcare professions can help by establishing a diagnosis, managing chronic conditions and advocating on behalf of the client and caregiver. Respite services, such as home care and day programs, give caregivers time to recharge, spend time with their family, and maintain employment. As the client’s condition deteriorates, however, caregivers may struggle to maintain the patient’s safety and quality of life. In these cases, long-term placements in facilities serve as last resort solutions that provide immense relief to caregivers
4.4 Distress Lines	Distress lines available 24 h a day are helpful in supporting and providing referrals to those who are acutely overwhelmed with the responsibilities of caregiving
4.5 Non-profit associations	Not for profit associations were noted by caregivers for: Reinforcing and supplementing healthcare services and offering personalized solutions, resource referrals and emotional support. They can offer more in depth discussion of the illness, the implications and the care options available, and tend to spend more time with patients, offering more personalized solutions. *“I realized that the things that my wife was doing was not unusual. That I learned that other people were experiencing the same thing”* Connecting caregivers with community and system resources offering invaluable support groups, which provide a safe space for caregivers to talk about their experiences with others who share similar experiences. During these meetings caregivers have the opportunity to verbalize their struggles, hopes and fears. *“It is nice to know that when you are in these groups you are dealing with like*-*minded people who have the same problems”* In addition, members of the group can offer emotional support, solutions and coping strategies. *“I found the support groups just absolutely wonderful”* and “*I feel supported just by being able to have this community with other people and being able to share”*

*HCPs* health care professionals

### Caregiver experience: recommendations and priority research areas

The focus group participants made recommendations as to ways to better support them in their caregiving, including: supports and services, caregiver education, Health Care Professional (HCP) education, more and better access to resources, system navigators, a more responsive caregiver centered system, financial supports, and political advocacy. An elaboration of these themes with supporting quotes can be found in Table 4.

**Table 4:** Caregivers experience: recommendations and priority research areas

Themes	Findings and supporting quotes
Supports and services	Caregivers require supports and services, such as community resources, workshops, emergency management, placement options, and respite services. *“We need resources, placements, guides in order to let people know how to find the right accommodation that meets the needs of both the caregiver, and the individual who’s experiencing the struggles”* Support for understanding the implications of the diagnosis, preparing for the future, and addressing concerns not covered in a medical visit. Emotional support and advice is also beneficial in decreasing distress, improving coping abilities and increasing quality of life. Caregivers would like resources and supports to be in place at the time of the diagnosis. *“…to have these things done before the dementia is full blown where it’s totally out of your control and you’re in a situation like this. Pre*-*emptive resources would be wonderful”*
Public service announcements to helps caregivers recognize they are caregiving	Because caregivers informally provide care for family members, a caregiver might spend years providing care without self-identifying as a caregivers, believing instead that they are simply helping the person. Caregivers recommend public service announcements to clarify the definition of caregiving and caregiver supports available. *“I never realized until July of last year that I was even a caregiver. I thought I was a helper so I never related myself to being a caregiver”*
Education for family caregivers	Caregivers identified the need for more education for family caregivers, particularly tools to better advocate for their care partners. They would like to acquire the language to work together with HCPs for the wellbeing of their care partner. *“Assertive communication so that I could better deal with my husband and better advocate for him with healthcare professionals*
Education for healthcare professionals	Caregivers hope that with education, HCPs will have greater confidence and more compassion when working with people suffering from dementia. *“It should be the medical profession that should be getting the training to communicate to the patient and their caregivers. Reverse the responsibility”* Caregivers would like to see more training for healthcare professionals regarding: Recognition of and intervention for Delirium Recognition of different types of dementia Intervention strategies Needs of different patient demographics Decision-making capacity assessments Effective communication with caregivers
Use of accessible language when communicating with caregivers	Communication by healthcare professionals with caregivers could be improved. Although medical jargon makes communication between HCPs more effective, caregivers and care partners do not understand the terminology. *“The impression I get with doctors is that they are not talking to me on my level … I’ll sort of use the analogy when you get manuals for your TV, your PVR, it’s written by an engineer for engineers”* The use of more accessible language to convey the pathophysiology, rationale for management changes, prognosis and impact on the patient’s life is advisable. Caregivers, due to their concern for the patient’s wellbeing and safety, are keen to be informed of details regarding and updates to medical management in a timely manner
More responsive senior and caregiver centered system is desired	At times, the decision-making process in the healthcare system feels overly policy-driven and unresponsive to the needs of the person. While caregivers understand the need for objectivity in the healthcare system, more personalized care is desired. Caregivers would also like to see their input have greater impact on patient care
More resources are needed	Caregivers would like more effective and timely communications from HCPs regarding available resources at the time of diagnosis. Resources from the system and community are especially important at the start of the caregiving journey - when caregivers are uncertain of the prognosis, the implications of the illness, and its impact. *“I kind of wish that I’d maybe had resources available earlier … that would enable me to have a more understanding view of things”* They also noted that resources should be accessible online, in a centralized information system, and personalized to match caregivers’ needs
System navigators and dementia health link are needed	Navigating the system can be a frustrating experience, therefore caregivers would like to have a navigator / professional advocate to guide them, recommend resources, and advocate on their behalf. *“A navigator, you need someone to tell you how to get through the system”* and *“We went through the breast cancer, SA… had six chemos and thirty radiations, there was ten times more the resources for breast cancer than there was for early onset Alzheimer’s and I would tell you that breast cancer is a pimple on a rhino’s butt to this, I would go through it ten times compared to this”* Caregivers indicated that a specific help and health line would be beneficial. *“Health Link … but just for people who have Alzheimer’s and then they could help a little bit at least to make things a little bit clearer or even when you’re just desperate… Because I know Health Link, that’s not their primary concern, but it would be just great”*
More political advocacy	Caregivers would like HCPs to take on a stronger advocacy role aimed at raising the awareness of politicians to the challenges of caregiving, and advocate for more funding, support, and resources. *“We as people have to start telling our MLAs, our MPs”*
Financial support - expanded tax credits	Caregivers expressed the need for an expansion of the eligibility criteria regarding caregiver tax credits to include spouses. This would lessen the financial burden for families. *“There’s a caregiver tax credit and it only applies to siblings or children, even friends or neighbours who are providing care; but it does not apply to spouses. So if there was a form letter that I could put my name to and just sort of personalize it to describe my situation, to send it to them”*

*HCPs* health care professionals

## Discussion

The purpose of this paper was to describe family caregiver perspectives regarding barriers to and facilitators of their resilience, as well as offer recommendations on how the health care system can better support them. Caregivers identified several barriers to resilience, including increasing demands on their time and resources, changing roles and responsibilities within the family, challenges to learning about their care partner’s medical condition(s), their own emotional responses to providing care, financial strains, changing family and social dynamics, and personal health. Caregivers also identified several facilitators to resilience, including their motivations for providing care, a renewed sense of purpose and validation, spirituality, positive emotional experiences, and coping strategies. Overall, caregivers recommended that educational opportunities, including increased education for health care providers concerning delirium and dementia, increased access to resources, system navigators, financial supports, political advocacy, and a more responsive caregiver centered system would support family caregiving.

Canadian statistics speak to the staggering amount of informal health services caregivers provide (Sinha 2012). The types of care and amount of time spent on caregiving activities of the respondents mirror those represented in the 2012 survey (Sinha 2012). Unfortunately, providing care for family members can have an overwhelming effect on caregivers. Often, caregivers find themselves in untenable situations that jeopardize their own health, finances, family relationships and overall wellbeing. Left unclear about their care partner’s diagnosis, its implications, and uncertain future, family caregivers very frequently feel voiceless and powerless amidst the incredible responsibilities placed on them. These sentiments have been echoed in several recent reviews of family caregiving from Poland and Asia (Domaradzki 2015; Park and Park 2015; Chan 2010). In order to sustain caregiver support, programs and services are needed to explicitly address the needs of family caregivers and provide them with necessary supports to foster resilience and honour their contribution to care recipients and the system.

To foster resilience, caregivers require access to a wide range of community services and resources, including education, training in emergency care specific to the care partner, and respite supports. Importantly, caregivers express a need to better understand the minutia and implications of their care partner’s diagnosis to better prepare for the future. Medical supports can provide the caregiver with information regarding the diagnosis, how to manage the evolving nature of the chronic condition, and advocate on behalf of the caregiver and care partner. In addition, respite services can offer the caregiver opportunities to engage in important family moments with their spouses and children. This can help alleviate guilt associated with missing important events, allow the caregiver to recharge, and enable them to attend to employment needs.

Caregivers noted that resilience is also fostered when emotional supports are provided, resulting in decreased distress, improved coping, and increased caregiver quality of life. Additional emotional support can also come from the care partner. Resilience was enhanced when caregivers had an opportunity to cherish moments shared with their care partner, with one caregiver stating, “[I]f I can get her to smile, my day is made.” Friends and family are also important sources of instrumental support. They can alleviate burden by taking on responsibilities of caregiving, including the management of finances, housework tasks, and transportation, for the benefit of the care partner and primary caregiver.

Caregivers related a number of recommendations that could foster a supportive environment during their caregiving journey. Caregivers noted that advocacy skills and knowledge of medical jargon could enhance their ability to effectively interact with healthcare professionals. One caregiver identified the need for, “assertive communication so that I could better deal with my husband and better advocate for him with healthcare professionals.” In addition to personal education, caregivers would like more training for healthcare professionals on delirium and early dementia recognition and intervention. This training could lead to earlier diagnosis of patients and more timely access to system and community resources in disease progression. Due to the difficulty navigating the healthcare system, caregivers desired access to a system navigator and professional advocates to help them access resources and advocate on the behalf of the caregiver and care partner. Greater awareness of the caregiver experience is foundational to determining interventions to address caregiver needs.

## Limitations

This study reflects the results of a small sample of family caregivers of seniors with complex needs residing in one geographic area. Relatedly, participants self-selected to engage in the focus groups and provided information regarding their experiences as a caregiver. As a result, the discussion may be impacted and the findings may not be generalized across all family caregivers.

## Conclusion

Strategies to better identify caregivers and their needs would be invaluable to policy and decision makers, clinicians, and researchers. Inclusion of both family caregivers and the care partner in care planning can positively affect the resilience of family caregivers. As caregiver resilience is influenced by the care partner’s disease process, family and social dynamics, physical, social, emotional, spiritual and financial needs, life experiences, and resources and services available in the system, it is important to address these so that family caregivers can continue to provide much needed support to care partners beyond what the system can offer. A better understanding of the experience of family caregivers can inform the design of public policies, programs, and interventions for care of individuals with complex needs. This research study enhances our understanding of the experience and needs of family caregivers and provides evidence of the great need for research in this area.
